# The magnitude of pediatric mortality and determinant factors in intensive care units in a low-resource country, Ethiopia: a systematic review and meta-analysis

**DOI:** 10.3389/fmed.2023.1117497

**Published:** 2023-04-17

**Authors:** Misganew Terefe Molla, Amanuel Sisay Endeshaw, Fantahun Tarekegn Kumie, Tigist Jegnaw Lakew

**Affiliations:** ^1^Department of Anesthesia, College of Medicine and Health Sciences, Bahir Dar University, Bahir Dar, Ethiopia; ^2^Department of Statistics, College of Natural and Computational Science, University of Gondar, Gondar, Ethiopia

**Keywords:** pediatric, mortality, intensive care, factors, Ethiopia

## Abstract

**Background:**

Pediatric mortality after being admitted to a pediatric intensive care unit in Ethiopia is high when compared to high-income countries. There are limited studies regarding pediatric mortality in Ethiopia. This systematic review and meta-analysis aimed to assess the magnitude and predictors of pediatric mortality after being admitted to an intensive care unit in Ethiopia.

**Methods:**

This review was conducted in Ethiopia after retrieving peer-reviewed articles and evaluating their quality using AMSTAR 2 criteria. An electronic database was used as a source of information, including PubMed, Google Scholar, and Africa Journal of Online Databases, using AND/OR Boolean operators. Random effects of the meta-analysis were used to show the pooled mortality of pediatric patients and its predictors. A funnel plot was used to assess the publication bias, and heterogeneity was also checked. The final result were expressed as an overall pooled percentage and odds ratio with a 95% confidence interval (CI) of < 0.05%.

**Results:**

In our review, eight studies were used for the final analysis with a total population of 2,345. The overall pooled mortality of pediatric patients after being admitted to the pediatric intensive care unit was 28.5% (95% CI: 19.06, 37.98). The pooled mortality determinant factors were included the use of a mechanical ventilator with an odds ratio (OR) of 2.64 (95% CI: 1.99, 3.30); the level of Glasgow Coma Scale <8 with an OR of 2.29 (95% CI: 1.38, 3.19); the presence of comorbidity with an OR of 2.18 (95% CI: 1.41, 2.95); and the use of inotropes with an OR of 2.36 (95% CI: 1.65, 3.06).

**Conclusion:**

In our review, the overall pooled mortality of pediatric patients after being admitted to the intensive care unit was high. Particular caution should be taken in patients on the use of mechanical ventilators, the level of Glasgow Coma Scale of <8, the presence of comorbidity, and the use of inotropes.

**Systematic review registration:**

https://www.researchregistry.com/browse-the-registry#registryofsystematicreviewsmeta-analyses/, identifier: 1460.

## Introduction

According to the World Health Organization (WHO), acute pediatric critical illness is defined as “any severe problem with the airway, breathing, or circulation, or acute deterioration of conscious state; [which] includes apnea, upper airway obstruction, hypoxemia, central cyanosis, severe respiratory distress, total inability to feed, shock, severe dehydration, active bleeding requiring transfusion, unconsciousness, or seizures” (1). Children are frequently admitted to the intensive care unit (ICU), where advanced organ, hemodynamic, and airway support are required.

In recent decades, tremendously lower mortality and morbidity following admission to pediatric intensive care units (PICUs) have been reported in high-income countries due to medical care advancements (2). However, the mortality of children admitted to ICU remained higher in low- and middle-income countries such as Ethiopia (3). Most hospitals in low- and middle-income countries lack dedicated ICUs for pediatric patients, and there is a scarcity of trained medical specialists allocated to PICUs (4). In addition, essential drugs and equipment are not readily available (3, 5).

Although there are limited published data on mortality and its risk factors in critically ill pediatric patients admitted to PICUs in low-resource countries, few studies identified demographic, clinical, and physiological factors contributing to the death of patients in PICUs. Malnutrition, length of the PICU stay, multiorgan failure, shock, infection, mechanical ventilation, and vasopressor support are among the risk factors reported (6–11).

In Ethiopia, few studies are available concerning pediatric critical care outcomes and predictors of mortality in PICU. Using currently available evidence in Ethiopia, this systematic review aimed to study the incidence of mortality among patients admitted to PICUs and factors associated with mortality in PICUs. This review's findings can help stakeholders improve the critical care provided to the patients admitted to PICU by using evidence-based, more effective, and comprehensive treatments. It aids and simplifies allocating resources to patients who need them the most, depending on the risks they pose as identified by this review. In addition, this review will help fill the information gap and guide further pediatric and critical care studies. Therefore, this review aims to estimate the pooled magnitude and determinant factors for pediatric mortality in the intensive care unit of Ethiopia.

## Methods

### Eligibility criteria

#### Inclusion criteria

This review includes observational studies conducted in the Ethiopia population that investigated the incidence and/or mortality risk factors among pediatric patients admitted to PICU, published until 1 September 2022. The age group was between 1 month and 16 years, and studies conducted in English were included in this review.

#### Exclusion criteria

Studies conducted on the adult population (age ≥ 16), case reports/case series studies, systematic reviews, studies conducted outside Ethiopia, preprint, and articles without full text were excluded.

### Information source and searching process

PubMed, Google Scholar, and African Journals Online Databases were searched using the following combinations of keywords: (“pediatrics” OR “children” OR “child” OR “pediatrics”) AND (“ICU” OR “intensive care unit” OR “critical care unit”) AND (“mortality” OR “morbidity” OR “outcome” OR “survival”) AND (“Ethiopia”). We identified and recorded the article that was eligible for our review.

### Protocol and registration

**Figure 1:**
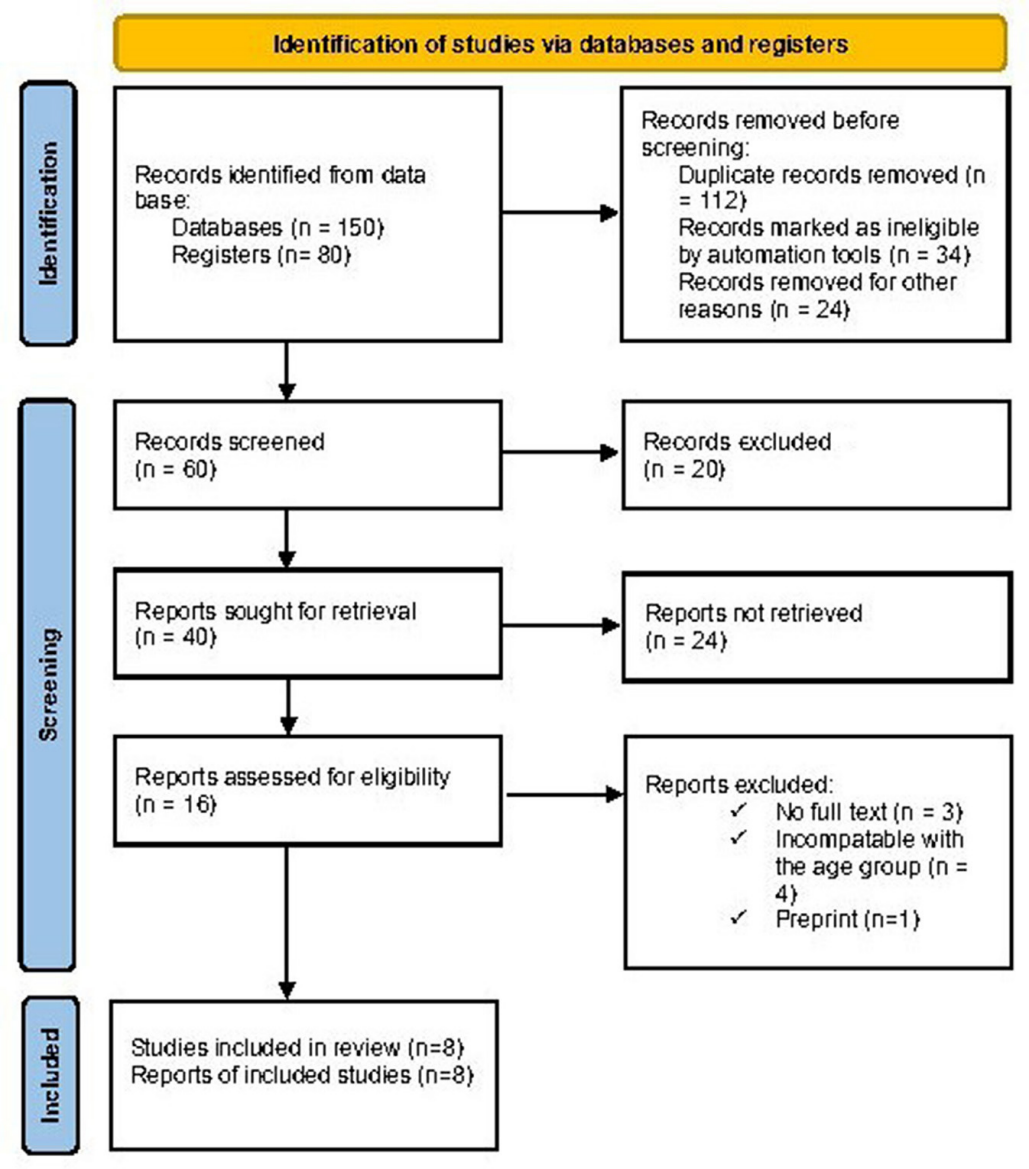
PRISMA flow chart.

This systematic review was conducted based on Preferred Reporting Items for Systematic Reviews and Meta-Analyses (PRISMA) guidelines (12). We formulated a search strategy and eligibility criteria using PRISMA guidelines before the review. The retrieved articles were then assessed based on pre-defined eligibility criteria. After finding relevant studies, data from each study were collected. This review was carried out in accordance with the Preferred Reporting Items for Systematic Reviews and Meta-Analyses (PRISMA) 2020 statement (Figure 1). This review was registered in the Research Registry with a unique identifying number of review registry 1460, and the link is https://www.researchregistry.com/browse-the-registry#registryofsystematicreviewsmeta~analyses/.

### Screening and selection process

In two stages, selected articles were identified. First, two authors reviewed titles and abstracts for eligibility, followed by full-text reviews. The other two reviewers extracted the data, and its completeness was cross-checked with each other who read the full text. When the two researchers disagreed, they openly discussed the disagreement and identified the inconsistency; all reviewers provided evidence and resolved the deviation based on scientific justification. Finally, after a thorough selection and screening of eligible articles, we obtained eight articles for analysis and reporting of this review.

### Study quality assessment

All authors checked the article's quality in retrieving each article for methodological quality that met the inclusion criteria in our review. All authors agreed on the quality of the studies. The methodological quality of our systematic review and meta-analysis compliance were evaluated using AMSTAR 2 criteria, and it was found to be of high quality (13).

### Outcome of interest

The incidence of mortality among pediatric patients admitted to PICU was the primary outcome of this systematic review. The mortality risk factors among pediatric patients admitted to PICU were reported as the secondary outcome in the studies that met the inclusion criteria.

### Data extraction and analysis

Using Microsoft Excel, eight articles that met the eligibility were retrieved from each study: the authors, publication years, study region, study period, sample size, study design, incidence, association measure, *P*-value, and mortality risk factors, including the use of a mechanical ventilator, use of inotropes, level of GCS < 8, length of ICU stay, presence of comorbidity, multiorgan dysfunction, medical case, weekend admission, and critical illness diagnosis data among pediatric patients admitted to PICU were selected. After entering the data, we transform it to Stata/MP version 17 (64-bit) software for analysis. A random effect meta-analysis was used for this review. DerSimonian and Laird's heterogeneity variance estimator was used, and a confidence interval was used for heterogeneity variance. The pooled magnitude of mortality and its determinants in pediatric patients admitted to PICU were analyzed as an overall pooled analysis and a factor analysis. The result was expressed using pooled mortality, along with its 95% confidence interval, *P*-value, and *I*^2^.

## Results

### Description of studies

During our systematic review, we included eight articles with a total sample size of 2,345 study subjects, which matched our objective. The final analysis included two articles from Addis Ababa, three from Amhara, two from Oromia, and one from the Tigray region (11, 14–20). All the studies were conducted from 2015 to 2022. One article was conducted with a cohort study design, while the other was conducted with a crossectional study design. In our review, the analysis method used for the three articles was chi-square analysis (14, 17, 20), while the analysis method for the five articles was logistic regression, and one article used the survival model (11, 15, 16, 18, 19, 21); it helps to minimize confounding factors, and this study includes the determinate factors for pediatric mortality in PICU (Table 1).

**Table 1:** Characteristics of included studies of pediatric patients admitted to a pediatric intensive care unit in Ethiopia.

**References**	**Study design**	**Study region**	**Publication year**	**Sample size**	**Death**	**Mortality in (%)**
Edae et al. (15)	Cross-sectional	Addis Ababa	2022	260	55	21.2
Tazebew et al. (14)	Cross-sectional	Amhara	2019	330	102	30.9
Seifu et al. (16)	Cross-sectional	Addis Ababa	2022	361	158	43.8
Ahmed (17)	Cross-sectional	Amhara	2022	305	72	23.6
Haftu et al. (18)	Cross-sectional	Tigray	2018	400	34	8.5
Teshager et al. (21)	Cohort	Amhara	2020	313	102	32.6
Bacha et al. (19)	Cross-sectional	Oromia	2022	206	59	28.6
Abebe et al. (20)	Cross-sectional	Oromia	2015	170	68	40

The characteristics of the determinant factors include the use of mechanical ventilators, length of stay, presence of comorbidity, and level of GCS < 8 were among those involved in the pooled analysis (Table 2).

**Table 2:** Characteristics of determinant factors of pediatric mortality in a pediatric intensive care unit, Ethiopia.

**Factors**	**Numbers of death**	**Percentage**	**Measure of association**	**95% CI**	***P*-value**	**References**
Level of GCS < 8	90	24.93	OR (10.5)	3.81–29.1	0.001	Seifu et al. (16)
Presence of comorbidity	127	35.18	OR (8.38)	3.42–20.5	0.001	Seifu et al. (16)
Use of mechanical ventilator	142	39.34	OR (11.2)	4.3–28.9	0.001	Seifu et al. (16)
Use of inotropes	99	24.43	OR (10.7)	4.09–27.81	0.001	Seifu et al. (16)
Patients admitted for medical case	112	50.9	OR (0.127)	0.37–0.41	< 0.05	Bacha et al. (19)
Weekend admission	31	23.66	HR (1.63)	1.02–2.60	< 0.05	Teshager et al. (11)
Use of mechanical ventilator	23	22.54	HR (2.36)	1.39–4.01	< 0.05	Teshager et al. (11)
Critical illness diagnosis	49	15.65	HR (1.79)	1.13–2.85	< 0.05	Teshager et al. (11)
Multiorgan dysfunction	97	44.09	OR (0.181)	0.08–0.41	< 0.05	Bacha et al. (19)
ICU stay duration 2–7 days	64	17.72	OR (7.27)	1.73–30.55	0.007	Seifu et al. (16)
ICU stay duration 7–14 days	43	11.91	OR (5.42)	1.18–24.80	0.029	Seifu et al. (16)
ICU stay duration 14–28 days	19	5.26	OR (7.02)	1.08–45.19	0.04	Seifu et al. (16)
Level of GCS < 8	6	16.2	OR (7.74)	1.1–54	0.04	Haftu et al. (18)
Use of inotropes	17	12.7	OR (10.4)	3.7–29	0.000	Haftu et al. (18)
Use of mechanical ventilator	6	37.5	OR (17.6)	2.2–14	0.007	Haftu et al. (18)
Presence of comorbidity	27	14.8	OR (10.2)	2.2–44	0.004	Haftu et al. (18)
ICU stay duration 2–7 days	16	6.6	OR (0.09)	0.023–0.39	0.001	Haftu et al. (18)
ICU stay duration 7–14 days	3	7	OR (0.094)	0.1–0.78	0.02	Haftu et al. (18)
ICU stay duration 14–28 days	1	6.7	OR (0.019)	0.01–0.45	0.01	Haftu et al. (18)

### The magnitude of pediatric mortality in PICU

**Figure 2:**
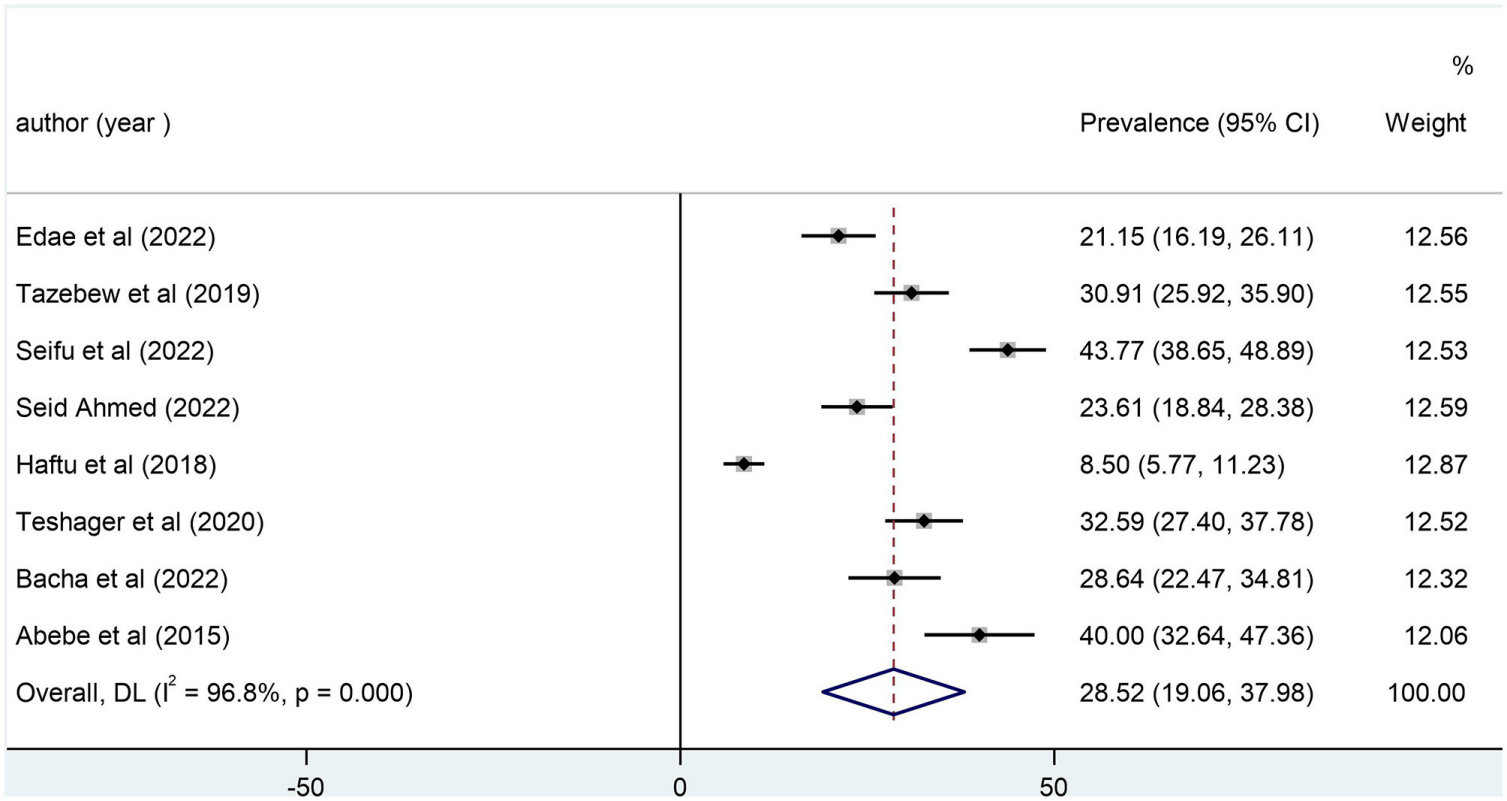
Pooled pediatric mortality.

In this systematic review and meta-analysis, the mortality rate of pediatric patients after being admitted to PICU was varying between the lowest, which was 8.5%, reported by Haftu et al. (18), and the highest, 43.8% reported by Bacha et al. (19). In our systematic review and meta-analysis, the overall pooled mortality rate among pediatric patients after being admitted to PICU was 28.5% (95% CI: 19.06, 37.98; Figure 2).

### Assessment of heterogeneity

**Figure 3:**
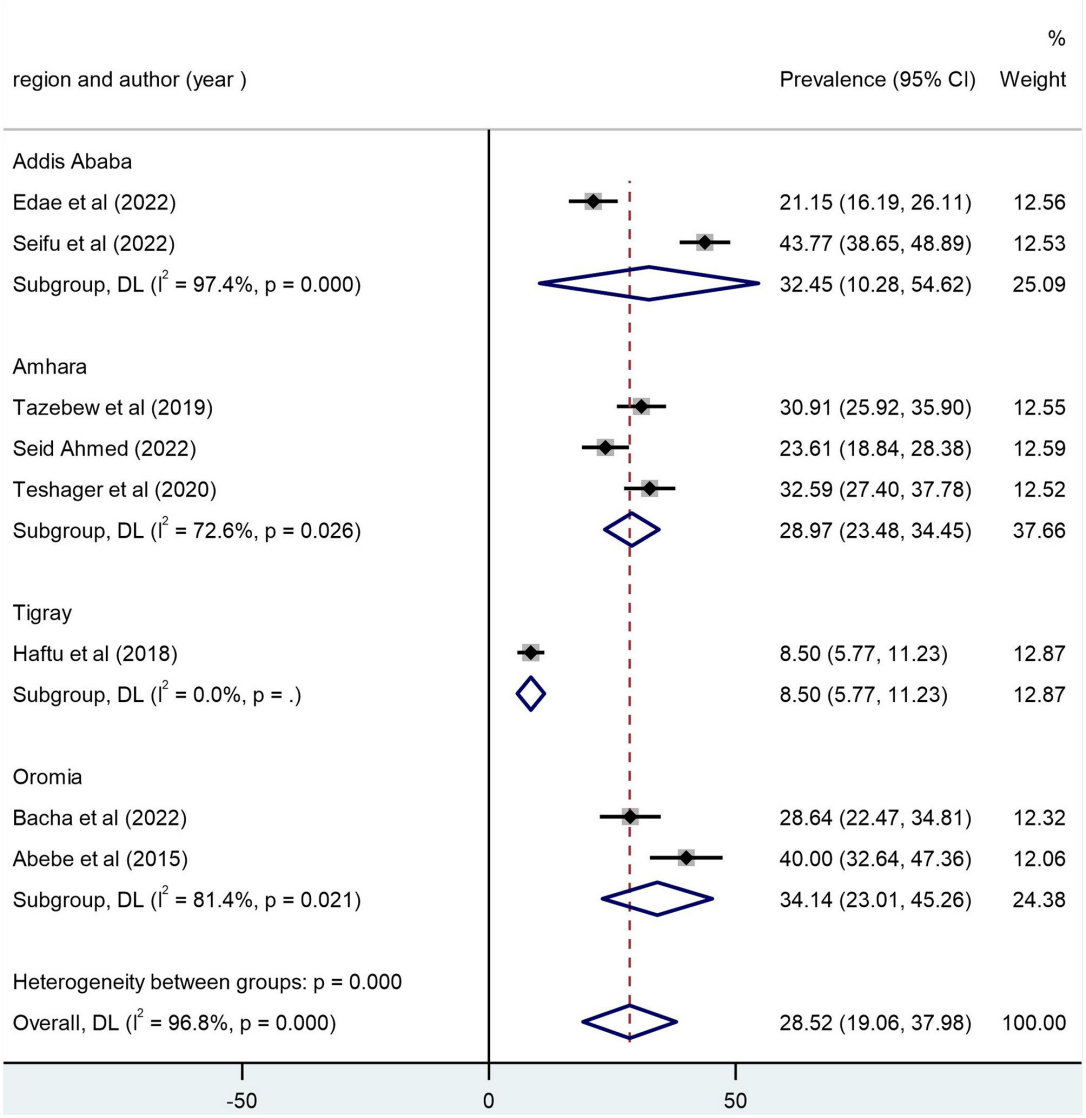
Subgroup analysis by sample size and region.

In this review, we used subgroup analysis to assess heterogeneity, and the *P*-value and *I*^2^ statistics were used to assess the heterogeneity between the studies. The *P*-value for a subgroup heterogeneity of 0.000 and the *I*^2^-test of heterogeneity of 96.8% were obtained. In order to assess heterogeneity between subgroups, we performed a subgroup analysis using the regional administrative state and the sample size of the included studies. From the subgroup analysis, the overall pooled mortality in Oromia was high with 34.14% (95% CI: 23.01, 45.26) and *I*^2^ = 81.4%, followed by a study conducted in Addis Ababa was 32.45% (95% CI: 10.28, 54.62) and *I*^2^ = 97.4%, a study conducted in Amhara was 28.97% (95% CI: 23.48, 34.45) and *I*^2^ = 72.6%, and a study in Tigray administrative state with an overall mortality of 8.50% (95% CI: 5.77, 11.23; Figure 3).

### Assessment of publication bias

**Figure 4:**
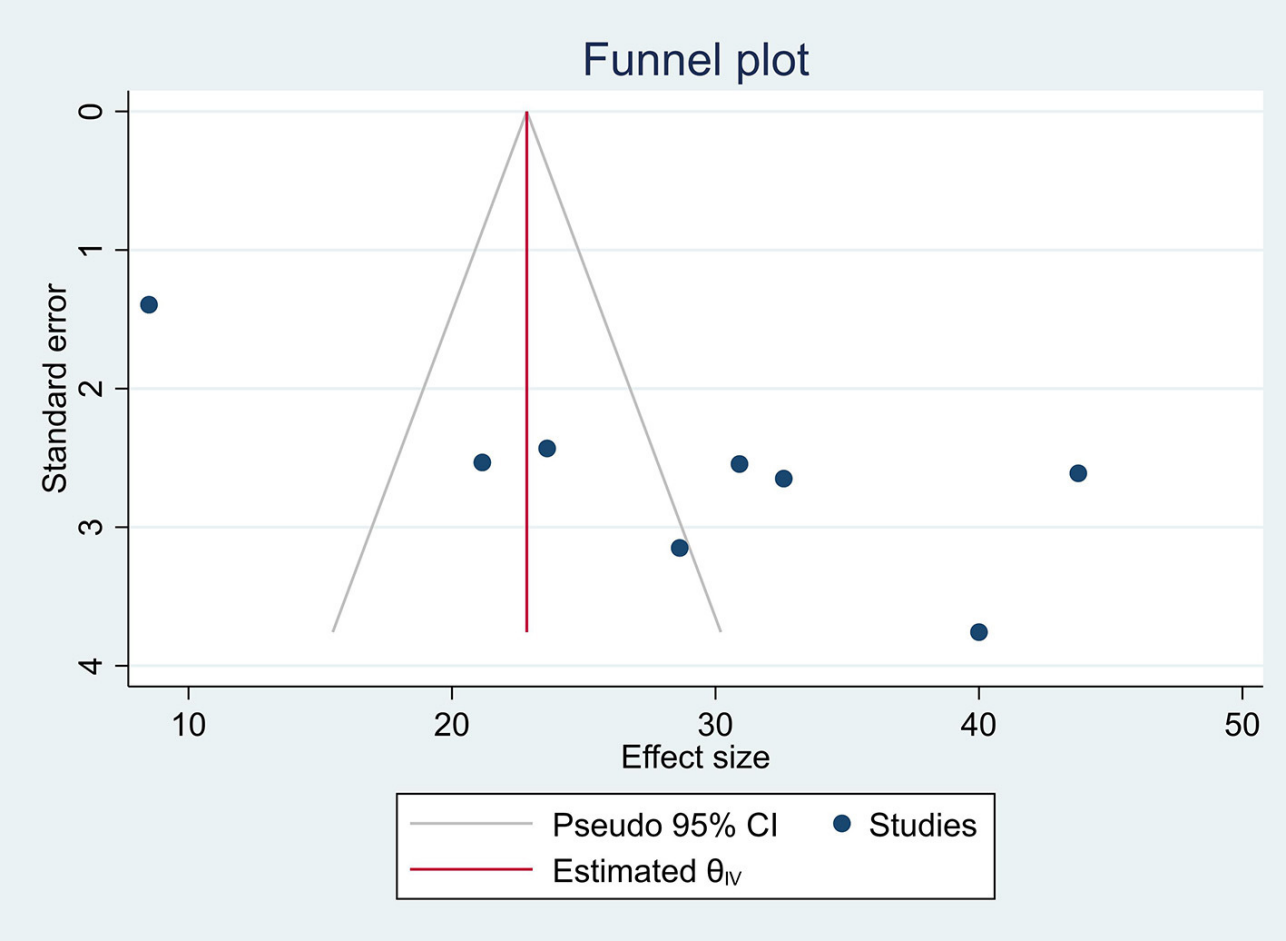
Funnel plot.

The funnel plot was used to check the publication bias and shows deviation from the symmetrical line. However, Egger's regression and Beggs correlation coefficients show a difference and a low possibility of publication bias with a *P*-value of 2.234 (Figure 4).

### Sensitivity analysis

**Figure 5:**
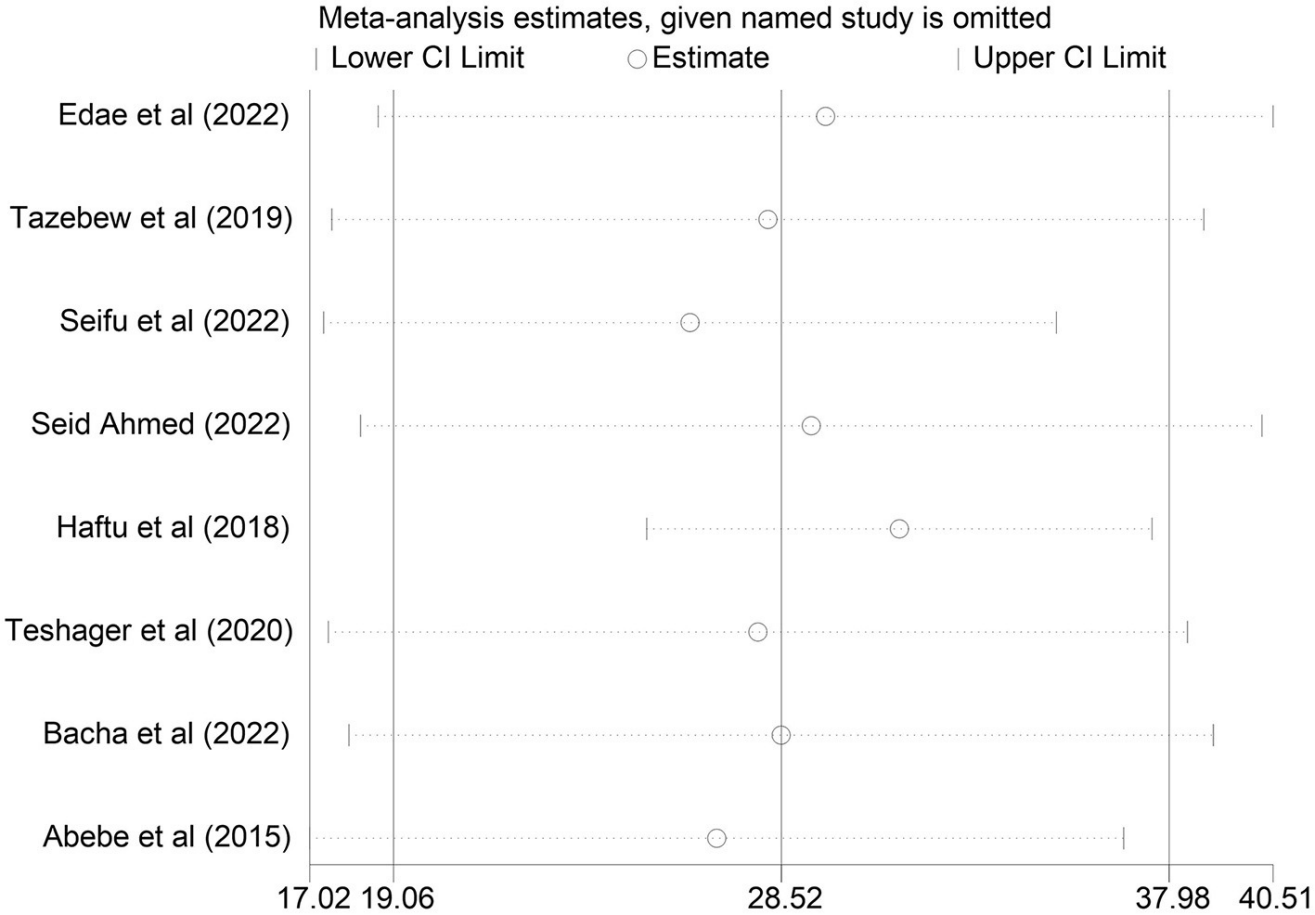
Sensitivity analysis.

We conducted a sensitivity analysis by removing a study with a lower and higher value on the review to show the effect of one study on the overall pooled summary effect, and we found that removing a study did not result in a significant summary effect on the overall outcome (Figure 5).

### Pooled analysis for determinant factors

#### Use of a mechanical ventilator

**Figure 6:**
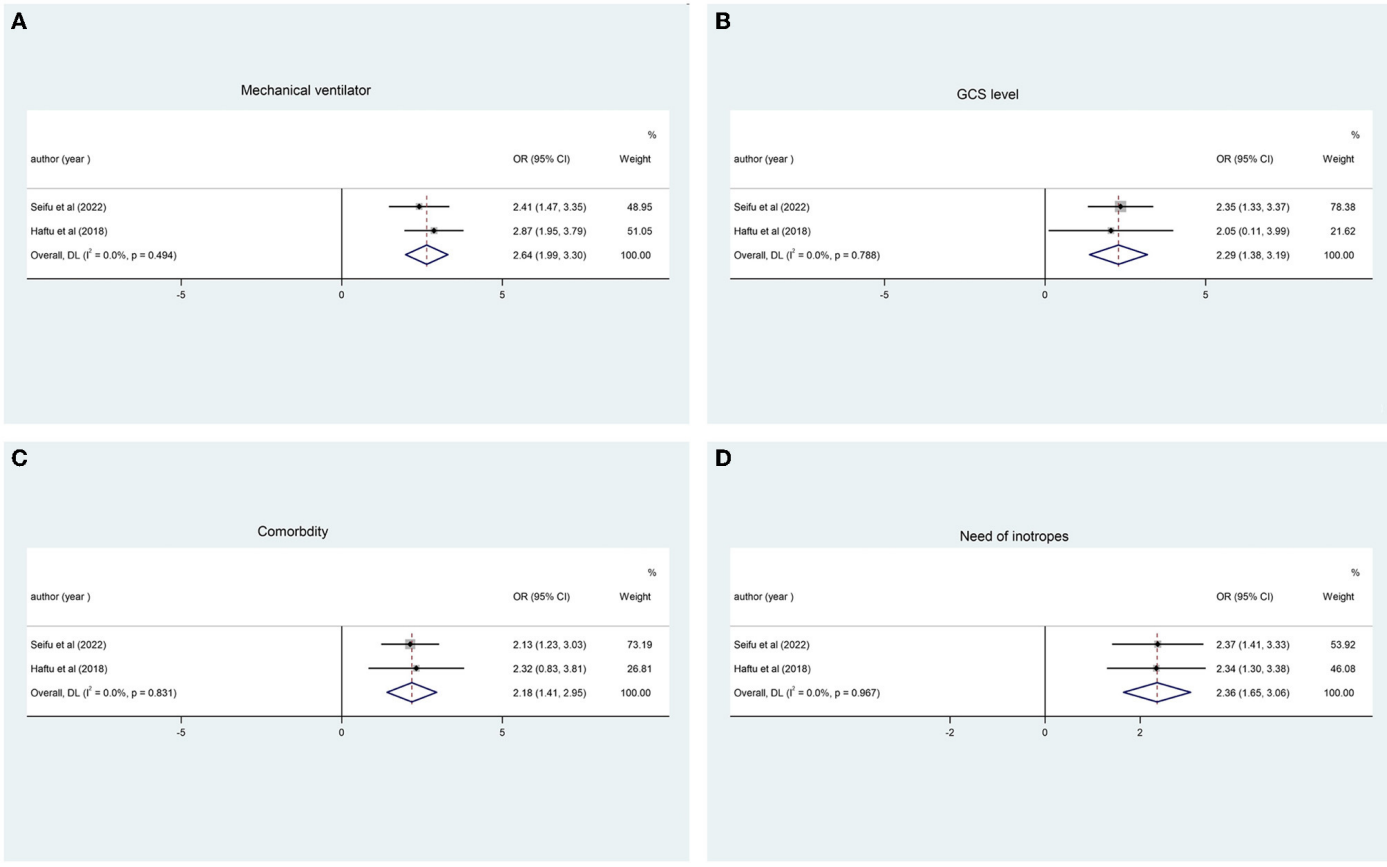
Determinant factors for pediatric mortality. **(A)** Use of a mechanical ventilator. **(B)** Level of GCS < 8. **(C)** Presence of comorbidity. **(D)** Use of inotropes.

The use of a mechanical ventilator for pediatric patients at PICU was the contributing factor stated by two studies (16, 18), with 2.64 times increases in overall pooled mortality in pediatric patients (OR, 2.64 CI: 1.99, 3.30; Figure 6A). Moreover, most patients required a mechanical ventilator due to shock, acute respiratory distress syndrome, and trauma.

#### Level of GCS < 8

The level of GCS of <8 was a factor for pediatric mortality in the PICU, as reported by two studies in our review (16, 18), with a pooled analysis of 2.29 times more likely to increase mortality (OR, 2.29 CI: 1.38, 3.19; Figure 6B). Considering that most patients brought to the ICU had low GCS of <8 and required intubation, it is a determinant for increased mortality.

#### Presence of comorbidity

In our review of pooled analysis, as stated by two studies (16, 18), the presence of multiple comorbidities increases the mortality of pediatric patients by 2.18 (OR, 2.18 CI: 1.41, 2.95; Figure 6C). In addition to the primary illness, comorbidities have been shown to have a significant impact on the clinical course, complications rate, and outcomes in the ICU, increasing the patient's criticality and physiologic derangement.

#### Use of inotropes

The pooled analysis review, stated by two studies (16, 18), shows 2.36 times more likely increase in the mortality of pediatric patients (OR, 2.36, CI: 1.65, 3.06).

Even if other determinate factors affect the mortality of pediatric patients, only the above factors are common in pooling analysis as determinate factors for pediatric mortality admitted to PICU (Figure 6D).

## Discussion

Studies conducted in Ethiopia on pediatric mortality admitted to PICU were high compared to high-income countries. In our review, the pooled mortality rate of pediatric patients after being admitted to the PICU was 28.5%. The use of a mechanical ventilator, level of GCS < 8, the presence of comorbidity, and the use of inotropes were the pooled determinant factors for the mortality of pediatric patients admitted to the PICU of Ethiopia.

The analysis of the article conducted on pediatric mortality at PICU in this review remains high. For instance, from two studies conducted in Addis Ababa, the pooled mortality of pediatric patients after being admitted to PICU was 32.45%. Three studies were conducted in the Amhara region, yielding a pooled mortality rate of 28.97%. Two studies were conducted in the Oromia region of Ethiopia, and the pooled mortality rate of pediatric patients was 34.14%. One study from the Tigray region of Ethiopia showed 8.5%. The difference in mortality among pediatric patients admitted to PICU was due to sample size difference, duration, and the number of studies included.

A study conducted on pediatric mortality after being admitted to PICU in low-income countries ranges from 6.7 to 51.1% (7, 22–25), but in developed countries, such as Europe and America, it ranges from 1.85 to 5.8% (26), and in Latin America, it reached up to 13.1% (27). The difference in the mortality of pediatric patients is due to the reason that there is limited availability of ICU setups, scarcity of trained staffing, and lack of availability of materials (14).

In our review, two articles stated that the use of mechanical ventilators was the factor for pooled mortality of pediatric patients at PICU. This review is supported by studies conducted in Jordan (23) and the Netherlands (28). The possible explanation is that most patients who require mechanical ventilators are more critical than those who do not, and it stays in for a couple of days, thereby increasing mortality. In addition, patients using a mechanical ventilator will be immobile for a prolonged time, raising the risk of venous thromboembolism. They are also more likely to acquire ventilator-associated pneumonia, which could lower their likelihood of survival (29).

The level of GCS of <8 has a factor in the mortality of PICU in our review. This finding is supported by studies conducted in Iran (30) and the Netherlands (31). The possible explanation for this factor was that decreased GCS level results in more critical patients and increased mortality. Another explanation could be that patients with low GCS have a higher mortality risk in the PICU due to the increased risk of hypoxia caused by compromised airway patency. In addition, cerebral hypoxia caused by hypotension in a decompensated illness process indicates multiorgan failure, which will have a serious impact on survival (32). In addition, the low GCS shows that the central nervous system is dysfunctional, which is the cause of the patient's slow recovery after being admitted to the ICU (33).

In our study, comorbidity was one of the determinant factors contributing to pediatric patients' mortality in PICU. This finding is supported by studies conducted in Jordan (23) and India (34). Comorbidities have been shown to have a significant impact on the clinical course, complications rate, and outcomes in the ICU. In addition to the primary illness, comorbidities increase the patient's criticality, and physiologic derangement is suggested as a possible explanation for the contributing factor of mortality (35).

In our study, the usage of inotropes medication in the PICU contributed to the mortality of pediatric patients. This study was supported by a study conducted in Indonesia (36). Since most of the ICU patients had severe illnesses and required inotropes, mortality was likely increased. The patient was admitted to the intensive care unit for persistent hypotension, who was resistant to volume replacement and experienced a refractory state and a worsening prognosis due to the administration of inotropes for a prolonged time and at a higher dose (37).

This review will provide information for further study, inquire about overall mortality, and identify the common determinant factors of pediatric patients' mortality admitted to the PICU. A limitation of this review is that a small number of studies were included in this review due to the limited availability of studies conducted in Ethiopia.

## Conclusion

Pediatric mortality admitted to Ethiopia's PICU is high compared to middle- and high-income countries. The determinant factors for pediatric mortality in PICU were the use of a mechanical ventilator, use of inotropes, level of GCS of < 8, and the presence of comorbidity. Therefore, there is a need for attention to reducing pediatric patients' mortality. Furthermore, this needs collaborative work with the policymaker, the government, the hospitals, and the health professionals working at PICU.

## Data availability statement

The raw data supporting the conclusions of this article will be made available by the authors, without undue reservation.

## Author contributions

MM and AE contributed to the conception of the review, writing of the review, interpretation of the literature based on the level of evidence, preparation, and critical review of the manuscripts. FK and TL interpreted the literature based on the level of evidence, preparation, and critical review of the manuscripts. All authors read and approved the manuscript.
